# TGFβ signaling pathways in human health and disease

**DOI:** 10.3389/fmolb.2023.1113061

**Published:** 2023-06-01

**Authors:** Pei-Yu Chen, Lingfeng Qin, Michael Simons

**Affiliations:** ^1^ Yale Cardiovascular Research Center, Department of Internal Medicine, Yale University School of Medicine, New Haven, CT, United States; ^2^ Department of Surgery, Yale University School of Medicine, New Haven, CT, United States; ^3^ Department of Cell Biology, Yale University School of Medicine, New Haven, CT, United States

**Keywords:** TGFβ, EndMT, aneurysm, cell fate, smooth muscle cell, endothelail cell, nanoparticle, RNAi

## Abstract

Transforming growth factor beta (TGFβ) is named for the function it was originally discovered to perform-transformation of normal cells into aggressively growing malignant cells. It became apparent after more than 30 years of research, however, that TGFβ is a multifaceted molecule with a myriad of different activities. TGFβs are widely expressed with almost every cell in the human body producing one or another TGFβ family member and expressing its receptors. Importantly, specific effects of this growth factor family differ in different cell types and under different physiologic and pathologic conditions. One of the more important and critical TGFβ activities is the regulation of cell fate, especially in the vasculature, that will be the focus of this review.

## 1 Introduction

### 1.1 Transforming growth factor beta (TGFβ) biology and signaling

#### 1.1.1 TGFβ isoforms in development and in adult tissues

TGFβ was first isolated from Moloney sarcoma virus-transformed fibroblasts as a polypeptide that could transform fibroblasts to form growing colonies in soft agar (Roberts et al., 1980). Since then, five subtypes of TGFβ (TGFβ1-5) have been identified. Of these, only TGFβ1,2, and 3 are expressed in mammals. The three mammalian TGFβ proteins have 70%–82% homology at the amino acid level, and a similar activity *in vitro* (Cordeiro et al., 2000). However, each TGFβ isoform has a distinct binding affinity for TGFβ receptors and appear to serve specific functions *in vivo* as shown by gene knockout studies. During development, all three TGFβ ligands are produced by a number of different cell types (Pelton et al., 1991; Derynck and Budi, 2019). A global knockout of the TGFβ1 gene during development in mice results in severe multiorgan inflammation and early death (Shull et al., 1992), while mice with a global TGFβ2 knock out die perinatally with multiple cardiac, craniofacial, cleft palate, non-cranial skeletal, eyes, inner ears, and urogenital developmental defects (Sanford et al., 1997). Mice with a global TGFβ3 deletion die within 20 h of birth, due to abnormal lung development and the presence of cleft palate (Kaartinen et al., 1995; Proetzel et al., 1995). Studies of global knockouts of TGFβ1 lacking T and B cells (Tgfb1^−/−^Scid^−/−^ mice) in adults showed delayed wound healing (Crowe et al., 2000). A TGFβ3 knock-in into the TGFβ1 allele resulted in mice that are viable but with reduced life span and defects in bone and tooth mineralization (Hall et al., 2013).

#### 1.1.2 TGFβ ligands synthesis and activation

Because of multifunctional nature of TGFβ signaling, its activation is tightly controlled at multiple levels from ligands production by various cell types to their release from binding proteins in the blood and in the extracellular matrix (ECM), to cognate receptors expression and activation, and, finally, to transcriptional complex formation and target gene expression. These multiple regulation steps are intended to ensure TGFβ signaling is activated and terminated in a timely fashion.

**FIGURE 1:**
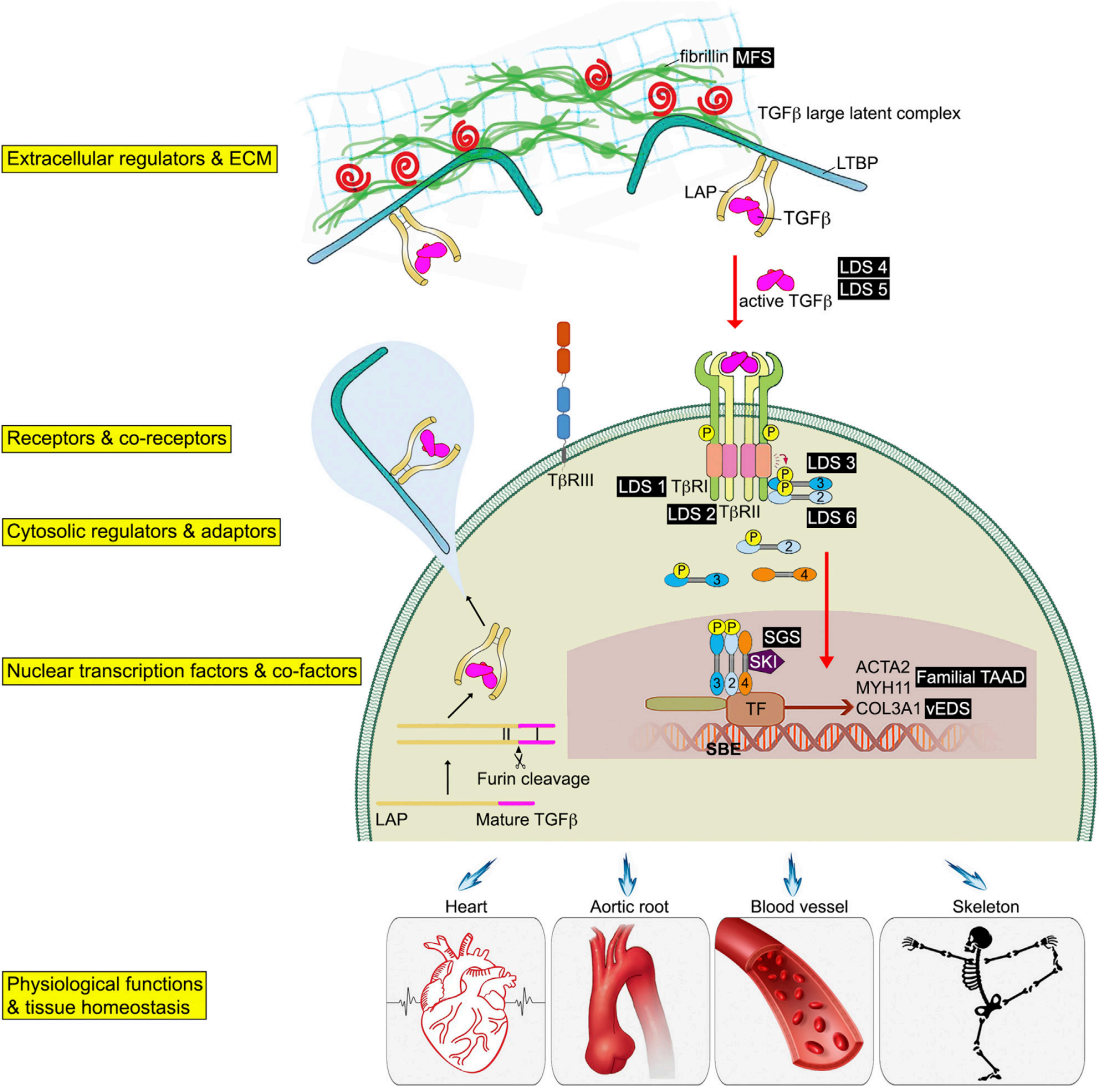
Schematic of the TGFβ signaling pathway associated genetic connective tissue disorders. TGFβ initiates signaling by assembling receptor complexes that activate SMAD transcription factors. Phosphorylated Smad2/3 proteins form a trimeric complex with Smad4, translocate into the nucleus, and regulate gene expression. Different connective tissue diseases associated with specific TGFβ signaling effectors are indicated in black boxes.

TGFβ ligands are initially synthesized as precursor proteins that undergo proteolytic cleavage. While many cell types have the ability to synthesize all three TGFβ isoforms, TGFβ1 is the predominant isoform under normal conditions in adult tissues (Assoian et al., 1983; Seyedin et al., 1985). However, TGFβ2 expression can increase many folds in endothelial cell undergoing endothelial cell to mesenchymal transition (EndMT) rendering it the most expression TGFβ ligand (Chen et al., 2012). Once synthesized, TGFβ ligands form disulfide dimers and are bound to the latency-associated peptide (LAP) (Gentry et al., 1988) (Figure 1). The LAP-TGFβ complex further interacts with the latent- TGFβ-binding protein (LTBP) through disulfide linkages to form the TGFβ large latent complex (LLC). When secreted into the extracellular space, LTBP remains noncovalently associated with LAP-TGFβ complex. LTBPs anchor the LAP-TGFβ complex to the ECM via interactions with fibrillin. Through interactions with LAP and LTBP, fibrillin-1 protein (encoded by the FBN1 gene) regulates the bioavailability of TGFβ and provides structural support in connective tissues (Robertson and Rifkin, 2016). Mutations in FBN1 can therefore lead to improper TGFβ sequestration, subsequently changing TGFβ availability and signaling, and ultimately resulting in development of Marfan syndrome (Arbustini et al., 2005). This highlights the importance of the ECM in maintenance of the connective tissue homeostasis, and how dysregulation of the ECM can play a key role in the development and progression of a number of human connective tissue disorders (CTDs). For this reason, we dedicate the entirety of Section 3 of this review to the discussion of TGFβ signaling defects-related heritable CTDs.

Although TGFβs are abundant in the ECM, they exist in the latent form and a LAP-TGFβ complex cleavage step is required for their release and activation (Miyazono et al., 1991; Rifkin, 2005). Several stimuli can release TGFβ from LAP complexes including direct proteolysis (such as the matrix metalloproteinases MMP2/MMP9), non-proteolytic dissociation mediated by thrombospondin-1 or integrins, as well as exposure to reactive oxygen species, or low pH (Munger et al., 1999; Yu and Stamenkovic, 2000; Annes et al., 2003). After being released from the LAP complex, a mature TGFβ can interact with and bind to TGFβ receptors, thereby initiating signaling. This reservoir of latent TGFβs in the ECM enables great regulatory plasticity and a rapid response to external signals.

#### 1.1.3 Regulation of TGFβ receptor activation

The initiation of TGFβ signaling starts with binding of a TGFβ ligand to its cognate serine/threonine kinase receptors on the cell surface (Wrana et al., 1992). These receptors are functionally categorized into two classes, type I and type II. Both type I and type II receptors have a similar structural organization and are evolutionarily closely related. Overall, there are seven type I receptors and five type II receptors in humans (Morikawa et al., 2016). These can form heterodimer complexes capable of binding various TGFβ ligands (Yamashita et al., 1994). In this review we will focus on the most common TGFβ receptors, TGFβR1 and TGFβR2 as these are the principle TGFβ receptors in the vasculature (Goumans et al., 2002; Carvalho et al., 2007; Nguyen et al., 2011). All three TGFβ isoforms bind to TGFβR2 and do not initially interact with TGFβR1 alone. Unlike TGFβ1 and TGFβ3 that have a high affinity for TGFβR2, TGFβ2 has a lower affinity thus requiring the presence of an additional type III TGFβ receptor (Cheifetz et al., 1990) (Figure 1).

**FIGURE 2:**
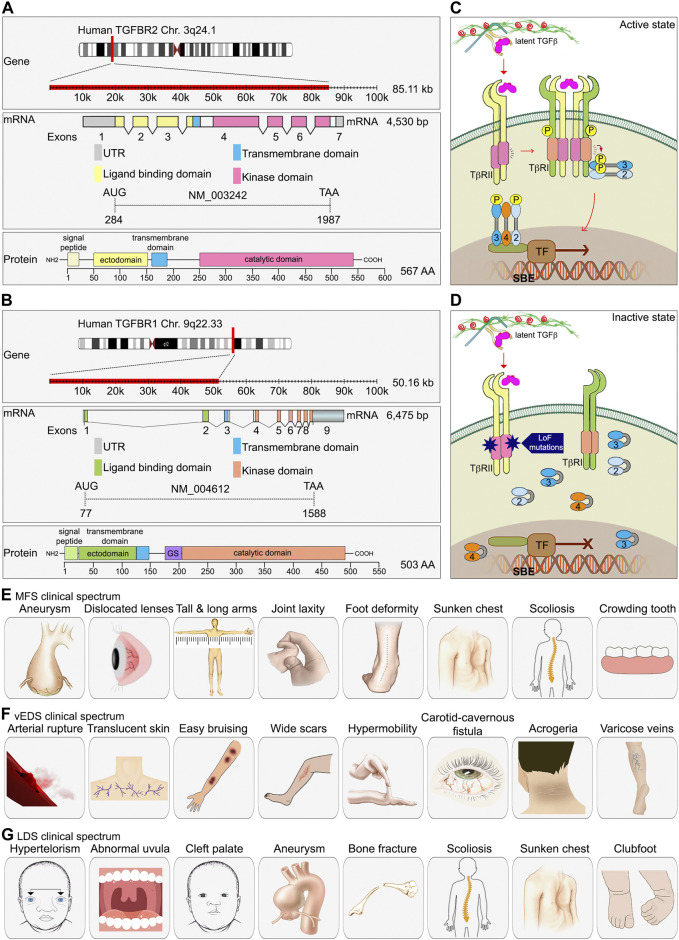
The essential role of TGFβ signaling in connective tissue disease development. **(A, B)** A schematic diagram of TGFBR1 and TGFBR2 gene, exons and protein domain organization. As compared to TGFBR2, TGFBR1 contains a unique glycine/serine (GS) domain followed by the kinase domain. **(C)** TGFβ signaling components. **(D)** In type 2 LDS, missense mutations found in the serine/threonine kinase domain of TGFβR2 lead to decrease TGFβ signaling. **(E–G)** Clinical spectrum of findings in MFS, vEDS, and LDS.

TGFβR2 is the most important and well-characterized type II TGFβ receptor. The gene, located on chromosome 3q24.1, is approximately 85 kb. Its mRNA transcript is 4,530bp long and contains 7 exons. The TGFβR2 protein is a 70/80 kD polypeptide with an N-terminal extracellular ligand binding ectodomain, a transmembrane region, and a C-terminal portion that contains the serine/threonine kinase domain (Wrana et al., 1994). Two such polypeptides combine to form a TGFβR2 homodimer that functions as a constitutively active serine/threonine kinase (Figure 2A).

The TGFBR1 gene is also known as Activin Receptor Like Kinase 5 (ALK5). It is approximately 50 kb long and located on chromosome 9q22.33. The RNA transcript is 6475 bp long and contains 9 exons. Similar to TGFBR2, TGFBR1 is also a homodimer made of two polypeptides with a molecular weight of 53 kDa. It has a hydrophilic extracellular region, a transmembrane domain, and an intracellular domain. The intracellular region near the plasma membrane contains a glycine/serine-rich domain (GS domain) that is associated with its autophosphorylation (Wrana et al., 1994). To prevent ligand-independent activation of TGFβR1 by TGFβR2, TGFβR1 interacts with FKBP12 (FK506-binding protein 12) and adopts a kinase inactive conformation (Chen et al., 1997; Huse et al., 1999). FKBP12 dissociates from TGFβR1 after TGFβR1 forms a complex with a ligand-activated TGFβR2. The schematic diagram of the TGFBR1 gene is presented in the Figure 2B.

In addition to TGFβ serine/threonine kinase receptor (TGFβR1 and TGFβR2), there are two type III TGFβ receptors, endoglin and betaglycan. Their main role is to increase the specificity of ligand-receptor-intracellular effector interactions and allow greater regulation of TGFβ signaling in specific cellular contexts (Kim et al., 2019). Endoglin was the first type III receptors to be identified. Both endoglin and betaglycan are membrane-anchored proteoglycans that contains a large extracellular domain involved in TGFβ ligand binding, a single pass transmembrane region, and a short intracellular domain that is involved in regulation of receptor trafficking (Figure 1). Due to the lack of a kinase domain, neither endoglin nor betaglycan play a direct role in the intracellular signaling. However, they bind all three TGFβ isoforms with a preference for TGFβ2. This leads to the retention of TGFβ ligands in the ECM and creates a ligands reservoir for facilitating their binding to TGFβR2. Upon ligand binding to the extracellular domain of the TGFβR2, the intracellular domain of TGFβR2 changes the structure conformation that allows a close interaction between TGFβR1. TGFβR2 kinase-mediated phosphorylation of the TTSGSGSG sequence of TGFβR1 GS domain leads to activation of TGFβR1. Activated TGFβR1 then phosphorylates downstream Smad transcription factors (Figure 2C).

#### 1.1.4 Canonical TGFβ signaling cascade- Smad proteins

The most studied and best-known intracellular effectors regulating the canonical TGFβ signaling cascade are Smad proteins. The name of “Smad” proteins comes from the fusion of names of two orthologous proteins: small body size (Sma) from *Caenorhabditis elegans* and Mothers against decapentaplegic (MAD) *from D. melangolaster* (Liu et al., 1996; Savage et al., 1996). The SMAD family consists of 8 members and is divided into three sub-families based on their structure and their roles in TGFβ signaling. One sub-family is that of receptor-regulated SMADs (R-SMADs) and includes SMAD1, 2, 3, 5 and 8. R-SMADs are substrates for the activated type I receptor. Once phosphorylated by the activated receptor, R-SMADs move to the nucleus and regulate expression of specific target genes (Massague, 1998). The second sub-family is made of a single common SMAD (co-SMAD) SMAD4. Co-SMAD interacts with activated R-SMADs facilitating their signaling. The last sub-family is made of inhibitory SMADs (I-SMADs) and includes SMAD6 and 7. I-SMAD antagonizes signal transduction that is mediated by R-SMAD and the Co-SMAD.

The involvement of an R-Smad in a particular TGFβ signaling event is tissue-specific and context-dependent. For example, activation of the type I receptor TGFβR1 results in phosphorylation of Smad 2 or 3 while activation of the type I receptor ALK1 results in phosphorylation of Smad 1, 5 or 8 (Schmierer and Hill, 2007). Smad4 is used by all members of the TGFβ-superfamily. The R-SMADs and SMAD4 are being continuously shuttled in and out of the nucleus even in the absence of the full TGFβ signaling pathway activation (Inman et al., 2002). Once activated, two R-Smads form a trimeric complex with one Smad4, that then translocates to the nucleus and binds to DNA at the palindromic sequence 5′-GTCTAGAC-3′ known as the Smad binding element (SBE) on the target gene. Once bound, R-SMADs interact with additional transcriptional co-factors to fine-tune expression of the target genes (Figure 1).

The level R-SMADs phosphorylation as well as their nuclear accumulation are frequently used as readouts of TGFβ pathway activity in mechanistic and functional studies. The inhibitory Smads SMAD6 and SMAD7 function as negative regulators of TGFβ signaling. Besides the I-SMADs, SKI (Sloan-Kettering Institute Proto-Oncogene) and SKIL (SKI like proto-oncogene) also bind directly to SMAD2, 3 and 4 and recruited to SBE sites. This interaction blocks the ability of R-SMADs to activate gene expression (Luo et al., 1999; Stroschein et al., 1999) (Figure 1).

In addition to the canonical Smad-mediated signaling, TGFβ can also activate non-canonical signaling cascades that include MAPK pathways, such as the ERK, JNK, and p38 MAPK, as well as small GTPases-dependent pathways under some physiological and pathological conditions (Zhang, 2009).

## 2 Cell type-specific effects of TGFβ signaling in cell fate determination

Since the discovery and characterization of TGFβ cytokines during the 1980s, research conducted over the following decades had defined core molecular components of the pathway. However, it became evident that TGFβ can exert different, sometimes opposite cell fate effects depending on a cell type and specific tissue contexts. For example, while TGFβ inhibits proliferation of epithelial cells, it promotes growth of fibroblasts (Tucker et al., 1984; Strutz et al., 2001); it inhibits tumor development at early stages and drives tumorigenesis at later stages (Cui et al., 1996). It also became clear that TGFβ had a different, difficult to define effect on development and progression of atherosclerosis. This had led to a detailed look at the growth factor’s effect on different vascular cell types.

**FIGURE 3:**
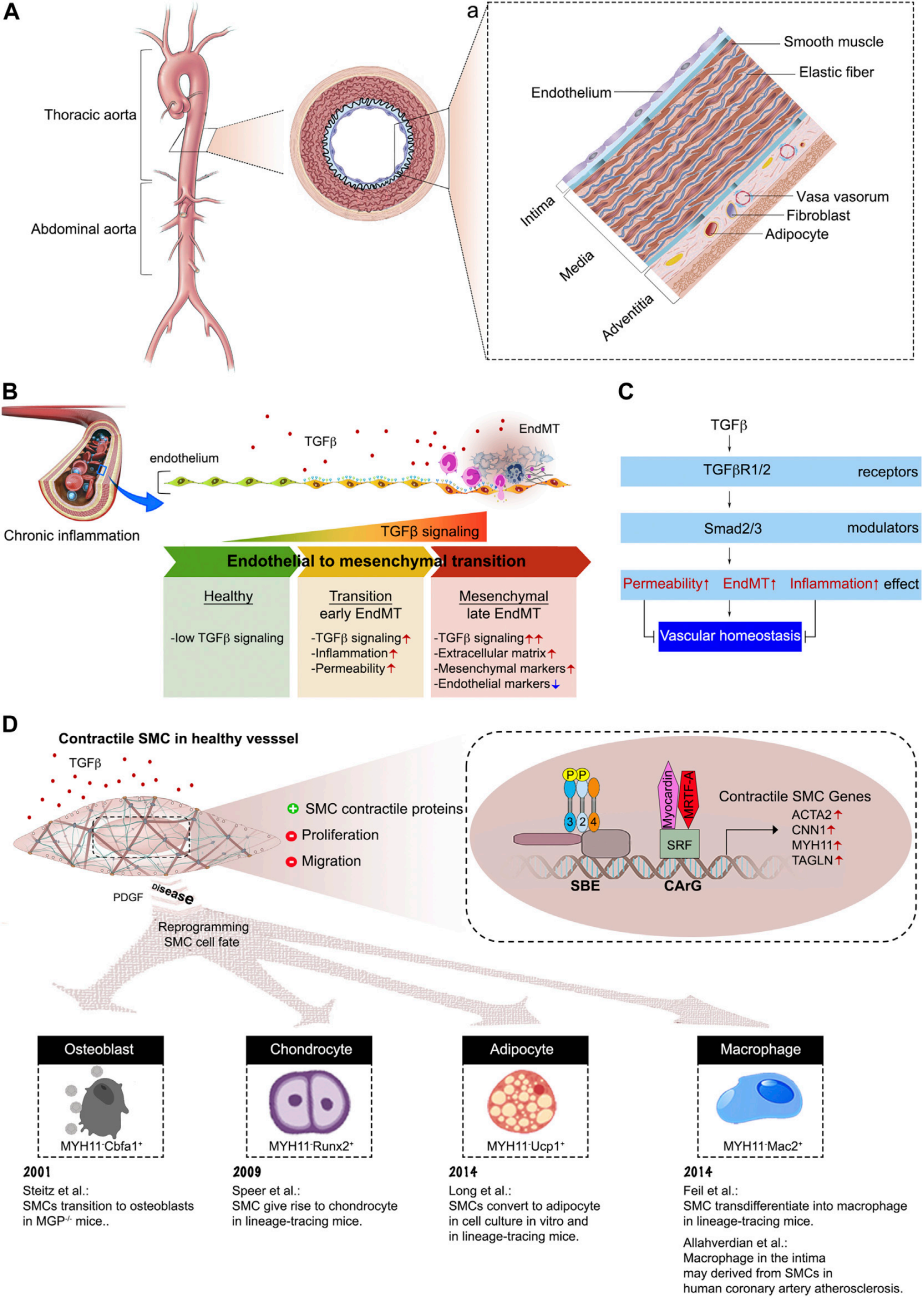
The composition of aortic vessel wall. **(A)** Cross-sectional view of the aorta, with the boxed region showing the position of the vessel wall enlarged in panel **(a)**. **(a)** A schematic of the three vessel layers: intima, media, and adventitia. Each layer has a unique cellular and extracellular matrix composition. **(B)** Endothelial cells undergo endothelial-to-mesenchymal transition in response to TGFβ stimulation. **(C)** Endothelial cell TGFβ/Smad signaling disrupts vascular homeostasis. **(D)** Summary of SMC cell fate transitions.

The arterial wall is a complex and surprisingly dynamic structure characterized by tightly regulated interactions between structural proteins (e.g., elastin and collagen) and a number of different cell types that make it up. All arterial walls have the same triple layer composition that include intima, media and adventitia (Figure 3A). The intima is formed by a single layer of endothelial cells (ECs). Adhesion and tight junctions between ECs prevent blood from leaking into the wall of the blood vessel. Importantly, ECs provide a smooth, non-thrombogenic surface for the blood flow and are involved in maintenance of vascular hemostasis, coagulation, and blood pressure. The medial layer is composed of smooth muscle cells (SMCs) and elastic fibers. The medial SMCs are aligned in the circumferential direction to enable modulation of vessel diameter and, hence vascular resistance and blood pressure. The adventitial layer contains collagen-rich extracellular matrix, fibroblasts, adipose tissue, and various stem and progenitor cells. The main function of the outer layer is synthesis and deposition of ECM components, allowing other cells to settle and migrate along this three-dimensional support. Finally, there is an expansive network of vasa vasorum, a vascular network that provides oxygen and nutrition to the arterial wall (Mulligan-Kehoe and Simons, 2014) (Figure 3A).

Various vascular insults induce two principal changes in the arterial wall structure: narrowing, potentially leading to a complete obstruction of the arterial lumen as occurs for example during the development of atherosclerosis, and weakening of the vessel wall, resulting in local vessel dilation and, potentially rupture as occurs during aneurysm formation. Both of these processes, lumen narrowing and aneurysm formation, occur due to specific TGFβ-induced changes in EC and SMC cell fates that are discussed in depth below.

### 2.1 TGFβ and the endothelial cell fate

Recent studies, including advances in mouse genetics lineage tracing, have completely changed our understanding of endothelial biology. The endothelium is now regarded as highly specialized cell type that exhibits organ-specific heterogeneity and maintains vascular integrity, homeostasis, and circulatory function (Aird, 2007a; Aird, 2007b; Ricard et al., 2021). The heterogeneous tissue-specific characteristics of ECs allow them to retain the capacity to drive tissue regeneration and repair in response to diverse biochemical and hemodynamic stimuli during development, adulthood, and adverse pathological situations especially cardiovascular diseases (Krenning et al., 2016). ECs also play a critical role in regulation of leukocyte adhesion and immune cell trafficking both under normal, and, especially, inflammatory conditions (Vestweber, 2015; Goswami and Vestweber, 2016; Arif et al., 2021). Whilst most cell types need TGFβ signaling for normal function, ECs are a very important exception with normal ECs having essentially no ongoing TGFβ signaling input. The key element of EC biology that make them insensitive to TGFβ is a very low level of TGFβR1 expression in normal adult arterial ECs (Ota et al., 2002; Goumans et al., 2003). The gain of TGFβ sensitivity, due to increased TGFβR1 expression, results in the development of endothelial-to-mesenchymal transition (EndMT), a process that represents a fate transition from endothelial to a mesenchymal-like phenotype (Chen et al., 2012) (Figures 3B, C). As the result of EndMT, ECs acquire expression of a variety of mesenchymal markers and leukocyte adhesion molecules that facilitate vascular wall inflammation. In fact, recent studies have shown that EndMT plays a key role in the development of atherosclerosis (Chen et al., 2015; Evrard et al., 2016), vascular graft failure and arteriopathies (Chen et al., 2012; Cooley et al., 2014), pulmonary arterial hypertension (Good et al., 2015; Ranchoux et al., 2015) and vascular aging (Fleenor et al., 2010) among other conditions.

### 2.2 TGFβ and the smooth muscle cell fate

SMCs play important roles not only in the basic physiological functions of a healthy blood vessel but are also critically important during disease development. In a healthy adult arterial vasculature SMCs are fully contractile cells that express a range of contractile and related cytoskeletal proteins that enable them to control the vascular tone. Furthermore, normal arterial SMCs are largely non-migratory and exhibit very low rate of proliferation (Neese et al., 2002) (Figure 3D). However, unlike cardiac and skeletal muscle cells, SMCs are not terminally differentiated and, in fact, retain the capacity to de-differentiate to a “synthetic” phenotype, functionally characterized by increased ability to migrate and proliferate. At the molecular level, this phenotype change is associated with reduction in expression of contractile/cytoskeletal proteins and increased expression of non-muscle proteins and matrix degrading enzymes (Rensen et al., 2007). Under certain disease conditions, the process of the SMC cell fate transition may advance much further leading to a near-complete loss of contractile protein expression and the appearance of expression of “mesenchymal” markers including those of fibroblast- macrophage- and osteoblast-like genes among others (Steitz et al., 2001; Speer et al., 2009; Allahverdian et al., 2014; Feil et al., 2014; Long et al., 2014). This phenotypic change, SMC-to-mesenchymal transition, allows SMC to increase the generating of specific ECM proteins, undergo proliferation and acquire migratory properties, all functions needed in injury-induced vascular remodeling situations.

Not surprisingly, regulation of SMC phenotypic plasticity is a highly complex process influenced by many factors. Among those, TGFβ is the key regulator and continuous TGFβ signaling input is an absolute requirement for the maintenance of the SMC contractile phenotype and of the structural integrity of the vascular wall (Li et al., 2014; Hu et al., 2015) (Figure 3D). TGFβ induces contractile gene expression by maintaining SRF (serum response factor) transcription and promotion of SRF-Myocardin binding to CArG (CC(A/T)6 GG) elements of contractile gene regulatory elements (Wang et al., 2004). Subsequent coordinated activity of the SMAD and SRF complexes increases expression of SMC contractile genes, such as ACTA2, CNN1, MYH11, and TAGLN.

In opposition to TGFβ, PDGF (platelet-derived growth factor) is the prime promoter of the synthetic phenotype in SMCs. PDGF induces KLF4 (Kruppel-like factor 4), a pluripotency associated transcription factor, which can disrupt SRF-Myocardin complex formation (Liu et al., 2005). As a result, PDGF causes rapid and sustained downregulation of contractile marker expression, in addition to activation of SMC proliferation and migration. In normal arteries PDGF levels are low or undetectable but can increase substantially in pathological states (Walker et al., 1986). This extreme plasticity of SMC biology is important in vascular injury repair, but also sets the roadwork for hijacking this process by various pathogenic stimuli thereby directly contributing to pathogenesis of some of the most common cardiovascular diseases.

## 3 Heritable connective tissue disorders associated with abnormal TGFβ signaling

TGFβ signaling provides an important link between the composition of the ECM and the vascular SMC function. Loss-of-function mutations in TGFβ signaling pathway components have been described in association with changes in aortic SMC phenotypes leading to structural changes to the aorta and causing heritable connective tissue disorders (CTDs) (Figures 1, 2D). To date, there are no cures for hereditary aortic diseases and their management focuses on prevention of potentially life-threatening cardiovascular complications. Here, we will discuss some of the CTDs including Marfan syndrome (MFS), Ehlers-Danlos syndrome (EDS), Loeys-Dietz syndrome (LDS), Shprintzen-Goldberg syndrome (SGS), and Familial Thoracic Aortic Aneurysms and Dissections (Familial TAAD) (Figure 1).

### 3.1 Marfan syndrome

Marfan syndrome (MFS) is the most common heritable CTDs. It most often occurs as an autosomal dominant inheritance from a parent and up to 25% sporadic random mutations occur occasionally. The estimated prevalence of MFS is about 1 in 5,000 with no difference among gender, ethnicity or race (Judge and Dietz, 2005). MFS is named after a French pediatrician Dr. Antoine Bernard Marfan (1858–1942) who described a five-year-old girl with skeletal malformations in 1896.

MFS is a multisystemic disorder affecting the skeletal (disproportionate overgrowth, joint laxity, scoliosis), ocular (lens dislocation and myopia), and cardiovascular systems (aortic root aneurysm and dissection, mitral valve disease) (Dietz, 1993) (Figure 2E). Cardiovascular manifestations are the most important cause of morbidity and mortality in patients with MFS, because of occurrence of aortic tears and aneurysms. The most common ocular feature of the MFS is ectopia lentis (dislocation of the lens of the eye), a condition seen in approximately 60% of patients. Skeletal abnormalities are typically the most striking physical features of the MFS and include tall stature, disproportionately long limbs and digits, mild to moderate joint laxity, chest deformity, vertebral column deformity and a narrow, high palate with crowding of the teeth (Cook et al., 2015) (Figure 2E). Diagnosis of MFS is based on clinical signs and family history analysis. Abraham Lincoln may have had Marfan syndrome given his abnormally tall figure, signs of aortic valve insufficiency and a similarly tall and lanky appearance of his mother (GordonAbraham Lincoln--a medical appraisal, 1962).

The classic MFS is caused by mutations in FBN1 gene (Fibrillin-1), a major structural component of the ECM necessary for TGFβ binding and activation (Dietz et al., 1991) (Figure 1). In MFS patients, a number of FBN1 gene mutations lead to impaired fibrillin-1 synthesis and subsequently defective generation of the connective tissue. Abnormal fibrillin-1 fails to bind TGFβ, resulting in increased circulating levels and excessive and deleterious TGFβ activity in multiple cell types. In the vascular system, the dysregulated TGFβ contributes to the elastin breakdown in the aortic wall, leading to aneurysm formation while the exact underlying cause of bone abnormality in MFS is not fully understood (Loeys et al., 2013).

### 3.2 Ehlers-Danlos syndrome (EDS)

Ehlers-Danlos syndrome (EDS) was named after two dermatologist, Edvard Ehlers and Alexandre Danlos, who described some of the most important clinical features of the syndrome including joint hypermobility, skin hyperextensibility, and fragile tissue (Parapia and Jackson, 2008). EDS is a group of inherited CTDs, that is less common than the MFS, occurring in one of every 250,000 individuals. EDS is divided into thirteen types and is caused by abnormalities in the structure, production and processing of collagen. Although it is known that collagen production is TGFβ-dependent, there is no evidence that mutations in collagen genes affect TGFβ signaling pathway in a direct way.

Vascular Ehlers-Danlos syndrome (vEDS) is one of EDS subtypes and is considered the most severe type (Malfait et al., 2017). It is caused by mutations in the type 3 procollagen (COL3A1) gene which is the major collagen isoform in blood vessels (Pepin et al., 2000; Schwarze et al., 2001; Frank et al., 2015). These mutations cause weaknesses in the vasculature and the heart. Individuals with vEDS can present with dissections, aneurysms, and rupture of various arteries throughout the body when they are relatively young. Vascular repairs in these individuals are difficult due to the fragility of vascular tissues. Other criteria for vEDS include translucent skin, easy bruising, abnormal cutaneous scar appearance, small joint hypermobility, carotid-cavernous fistula, acrogeria (skin aging), and early-onset development of varicose veins (Pepin et al., 2000) (Figure 2F). Due to the risk of organ rupture, which is often deadly, the median life expectancy of vEDS patients is 40–50 years.

### 3.3 Loeys-Dietz syndrome

In 2005, two physicians, Drs. Bart Loeys and Hal Dietz, identified mutations within TGFBR1 and/or TGFBR2 in a cohort of patients with a phenotype similar to the MFS but without FBN1 gene mutations (Loeys et al., 2005). The disorder was named the Loeys-Dietz syndrome (LDS) to designate this rare type of CTD. LDS affects people of all ethnic backgrounds, both female and male (Tan et al., 2013). Patients with the LDS can have mutations in a number of genes that encode various TGFβ signaling components, including TGFBR1, TGFBR2, SMAD3, TGFB2, TGFB3, and SMAD2, leading to LDS type 1, type 2, type 3, type 4, type 5, and type 6, respectively (Figure 1). Mutations in TGFBR2 are more frequent (55%–60%) than other gene mutations (TGFBR1, 20%–25%; SMAD3, 5%–10%, TGFB2, 5%–10%; TGFB3, 1%–5%; and SMAD2, 1%–5%) (Meester et al., 2017). Most LDS type 1 and type 2 mutations are missense mutations found in the serine/threonine kinase domain of both receptors. These mutations lead to decrease the receptors’ ability to propagate signal to downstream effectors in response to TGFβ stimulation (Uike et al., 2013; Meester et al., 2017) (Figure 2D). Indeed, cultured osteoblasts isolated from LDS neonates showed profoundly diminished Smad2 phosphorylation in response to TGFβ stimulation (Dewan et al., 2015).

Patients with the LDS were initially misdiagnosed as having either MFS or vEDS syndromes because of the similarity in phenotype with those two other syndromes. The most specific features of the LDS are hypertelorism (widely-spaced eyes) and the bifid uvula and/or cleft palate as well as generalized arterial tortuosity with frequent ascending aortic aneurysms (Figure 2G). Cardiovascular complications in LDS patients tend to be more aggressive than in patients with either MFS or vEDS, leading to dissection and rupture at smaller arterial diameters and at younger ages. Approximately 75% of people with the LDS have skeletal abnormalities including bone fractures and reduced bone density, abnormal curvature of the spine (scoliosis), sunken chest (pectus excavatum) or protruding chest (pectus carinatum), and inward- and upward-turning foot (clubfoot) (MacCarrick et al., 2014; Guerrerio et al., 2022) (Figure 2G).

### 3.4 Shprintzen–Goldberg syndrome

Shprintzen-Goldberg syndrome (SGS) is characterized by craniosynostosis, skeletal changes including arachnodactyly (long, slender fingers and toes) and camptodactyly (bent fingers), scoliosis, joint hypermobility, and aortic aneurysms (Doyle et al., 2012). SGS patients show a considerable phenotypic overlap with MFS and LDS patients but present with additional manifestations, including intellectual disability and severe skeletal muscle hypotonia. Recently, v-Ski avian sarcoma viral oncogene homolog was identified as the causal gene for this rare CTD. As mentioned earlier, the SKI gene encodes a protein that plays an important role in the negative regulators of the TGFβ signaling pathway (Figure 1). SKI itself cannot bind to DNA, but SKI binds to the R-SMAD/SMAD4 complex, which subsequently binds to DNA and causes repression of transcription of TGFβ responsive genes (Luo et al., 1999).

### 3.5 Familial thoracic aortic aneurysm and dissection (FTAAD)

The thoracic aortic aneurysms and dissections (TAAD) syndrome represents a spectrum of aortic diseases exhibiting both sporadic and inherited forms. About 20% of the TAAD patients have a family history of the disease, referred to as familial TAAD (FTAAD). FTAAD is primarily inherited in an autosomal dominant pattern with variable ages of onset and clinical symptom expression. SMC-specific contractile gene mutations are the most common cause of FTAAD. In particular, ACTA2 (gene encoding the smooth muscle α-actin) mutations are responsible for 14% of patients with FTAAD (Guo et al., 2007) while MYH11 (gene encoding myosin heavy chain) mutations accounting for <2% of familial TAAD cases (Zhu et al., 2006).

## 4 Non-inheritable chronic vascular disease associated TGFβ signaling defects

Perturbation of TGFβ signaling not only contributes to inheritable CTDs but are also implicated in pathogenesis of a number of non-inheritable diseases such as cancer, fibrosis, atherosclerosis, and pulmonary hypertension among many others. Here we will discuss how vascular pathology due to dysregulated TGFβ signaling contributes to disease pathogenesis.

**FIGURE 4:**
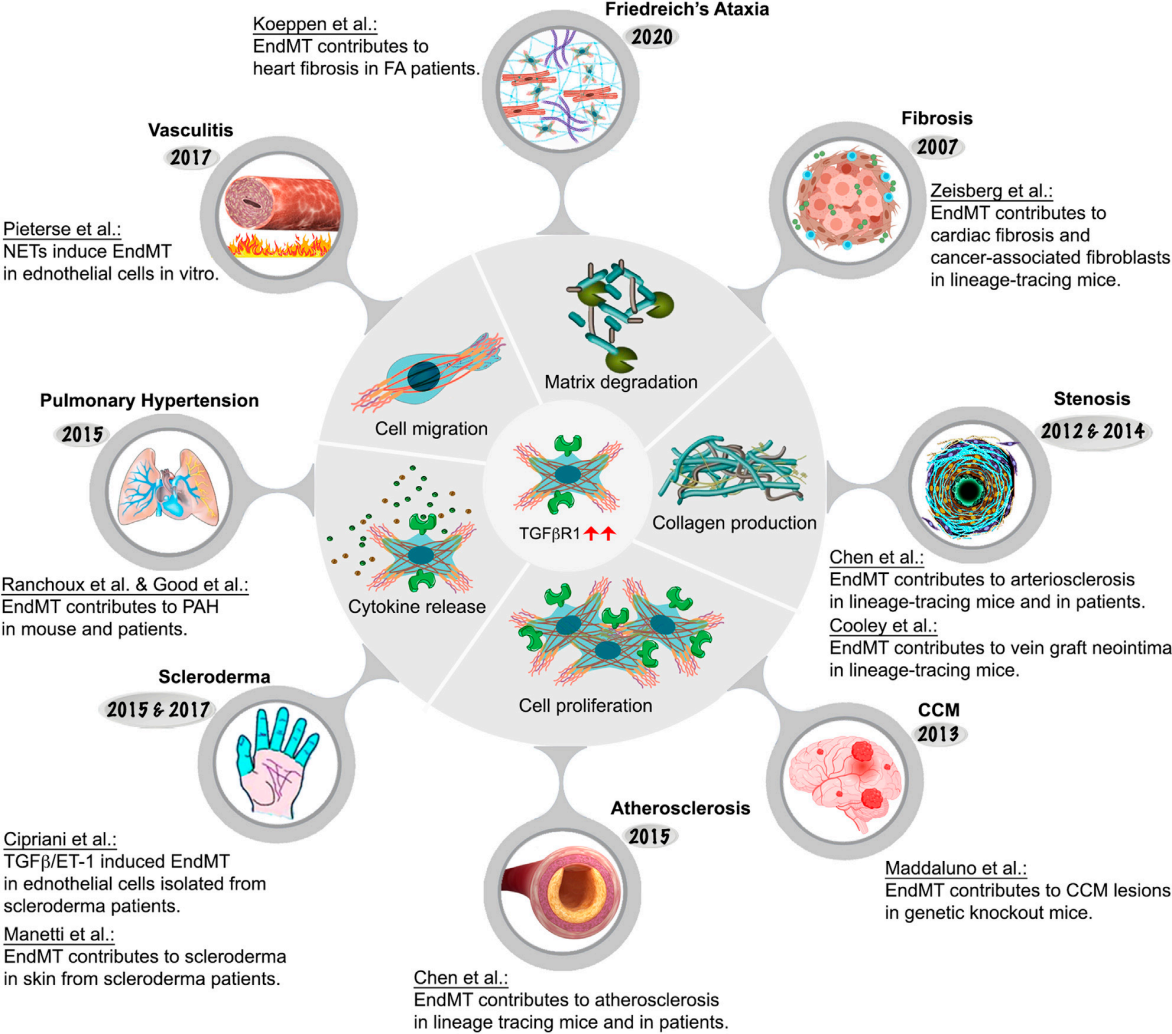
Endothelial-to-mesenchymal-transition associated diseases.

One of the perturbations of TGFβ signaling is EndMT, an important process that is essential during embryonic cardiac development (Kovacic et al., 2012) but that can also contribute to pathogenesis of a number of illnesses. As previously discussed, it involves endothelial cell fate transition to one of mesenchymal cell-like types (Dejana et al., 2017). While EndMT has been implicated in many adult pathological processes including fibrosis, stenosis, cerebral cavernous malformation, scleroderma, atherosclerosis, pulmonary hypertension, and vasculitis, among others, its very existence until recently remained a matter of intense debate as were its contributions to human pathology (Kovacic et al., 2019). As will be described in more detail later, from 2007 onwards EndMT research has grown exponentially due to advances in cell lineage tracing approaches in vascular biology. These advances shed new light on the origin of various cell types involved in in cardiovascular disease pathogenesis. Figure 4; Table 1 provide an overview of key EndMT studies in a chronological order.

**TABLE 1:** Summary of key EndMT studies.

Type of disease	Evidence of EndMT in affected tissues				
	Model of study	Tissue source	Method(s) to identify EndMT	EndMT inducer	Reference
Cardiac fibrosis	Mouse aortic banding	Heart	Tie1Cre lineage tracing, IHC	Hemodynamic stress	Zeisberg et al. (2007a)
Cancer fibrosis	B16F10 melanoma and Rip1Tag2 pancreatic islet tumor models	Melanoma and pancreas tissue	Tie2Cre lineage tracing, IHC, *in vitro* ICC	Gain of TGFβ signaling	Zeisberg et al. (2007b)
Arteriosclerosis	Mouse transplant rejection model, cardiac allografts with chronic rejection	Artery grafts, heart	Cdh5Cre lineage tracing, IHC, immunoblotting	Loss of FGF signaling	Chen et al. (2012)
Vein graft transplant	Vein grafting model	Vein grafts	Tie2Cre lineage tracing, IHC	Gain of TGFβ signaling	Cooley et al. (2014)
CCM	Mouse EC CCM1 KO, human CCM patients, *in vitro* ECs	Brain	Cdh5Cre lineage tracing, IHC, immunoblotting	Gain of Notch/BMP6 signaling	Maddaluno et al. (2013)
Atherosclerosis	Mouse atherosclerosis model, human coronary arteries, *in vitro* HUVECs	Large vessels	Cdh5Cre lineage tracing, IHC, immunoblotting	Loss of FGF signaling	Chen et al. (2015), Evrard et al. (2016)
Scleroderma	Scleroderma patients	Skin biopsies	IHC, electron microscopy, immunoblotting	Autoimmunity	Cipriani et al. (2015), Manetti et al. (2017)
Pulmonary hypertension	PH patients, MCT-induced PH in rats	Lung	IHC, electron microscopy, immunoblotting	Alterations in BPMR2 signaling	Good et al. (2015), Ranchoux et al. (2015)
Vasculitis	*In vitro* HUVECs	Endothelial cells	Immunoblotting	NET	Pieterse et al. (2017)
Friedreich’s Ataxia	FA patients	Heart	IHC	ROS	Koeppen et al. (2020)

### 4.1 EndMT and cardiac fibrosis

Cardiac fibrosis is characterized by an imbalance of the ECM production and degradation in the cardiac interstitium resulting in increased collagen deposition leading to myocardial stiffening, diastolic dysfunction, and eventually, heart failure. Cardiac fibrosis may occur in response to injury such as myocardial infarction or myocarditis or may be an independent disease process in its own right. Cardiac fibroblasts are traditionally viewed as the central cellular effectors in the pathogenesis of cardiac fibrosis (Ivey and Tallquist, 2016). They are of mesenchymal cells and their primary role involvers the regulation of the ECM turnover and cardiac interstitial space homeostasis, and facilitation of efficient communications between cardiomyocytes. They may also play a role in cardiac repair as seen, for example, after myocardial infarction when cardiac fibroblasts can and transform into cardiac myofibroblasts (Soliman and Rossi, 2020; Tallquist, 2020).

This traditional view of cardiac fibroblasts as cells of mesenchymal origin was challenged in 2007 when, using mouse EC-lineage tracing and marker protein staining, the laboratory of R. Kalluri suggested that a significant percentage of fibrotic cells in a mouse model of cardiac fibrosis had an endothelial origin (Zeisberg et al., 2007a). However, improved lineage tracing studies raised question regarding the existence of an EndMT process in cardiac fibrosis (Moore-Morris et al., 2014). At the heart of the controversy is the of Tie-1Cre reporter mice used to label and trace ECs. The constitutively active Tie-1Cre reporter demonstrates some degree of Cre recombinase activity in the hematopoietic lineage thereby confounding interpretation of the lineage tracing data. This study highlights the importance of using specific and faithful lineage tracing Cre-driver when examining phenotypic cellular transition and conclusions need to be interpreted cautiously. Another controversy is the lack of specificity of FSP1 (fibroblast specific protein 1) marker that was used to identify fibroblasts since other cell types, including immune cells, ECs, and SMCs can also express FSP1.

While endothelial involvement in cardiac fibrosis has remained unresolved, the process of EndMT has been shown to significantly contribute to cancer progression by playing a key role in generation of carcinoma-associated fibroblasts (CAFs) (Zeisberg et al., 2007b). CAFs are involved in tumor progression and can alter the tumor microenvironment in part through the release of tumor promoting cytokines, such as TGFβ and vascular endothelial growth factor (VEGF).

### 4.2 EndMT and vascular stenosis

Vascular wall healing occurs in response to injury such as surgical interventions, vascular grafting, intravascular mechanical injury or immune-mediated injury following organ transplantation. The injury insult triggers a pronounced SMC proliferation and subsequent SMC migration to the neointima that can cause narrowing of the involved vessels. Although neointimal SMCs have long been thought to be derived from phenotypic switching of SMCs migrating from the underlying media, there is increasing evidence that a substantial population of intimal SMCs is derived from the endothelium via the EndMT process. A study by Chen et al. using a mouse arteriosclerosis model shows that SMCs contributing to the neointima formation after arterial transplantation are primarily derived from the endothelium (Chen et al., 2012). Importantly, hematopoietic progenitors rarely contribute to SMCs in neointima formation (Chen et al., 2012). Rigorous mice lineage tracing studies showed that 30%–70% of EndMT cells within neointima belong to the mesenchymal lineage, becoming almost indistinguishable from SMCs, mesenchymal cells, and fibroblasts (Chen et al., 2012). This work was extended by Cooley et al., 2014 who implicated EndMT in the neointimal hyperplasia during vein graft remodeling (Cooley et al., 2014).

### 4.3 EndMT and cerebral cavernous malformation

EndMT involvement has been implicated in the development of cerebral cavernous malformations (CCM), a form of vascular dysplasia in which blood vessels in the brain present an irregular, thin-walled structure (Maddaluno et al., 2013). This condition causes vascular leakage and may result in a fatal cerebral hemorrhage. Endothelial cells in these vascular lesions express a set of mesenchymal (Slug, N-Cadherin, SM α-actin, Id1) and stem cell markers (Sca1, CD44) not typically associated with the normal endothelium (Maddaluno et al., 2013). Furthermore, ECs extracted from Ccm1 knockout mouse brains indicated that this altered gene expression was dependent on TGFβ and BMP signaling. However, other studies challenged the role of EndMT in CCM development and the issue remains unresolved (Jenny Zhou et al., 2016; Zhou et al., 2016).

### 4.4 EndMT and scleroderma

Systemic sclerosis (scleroderma, SSc) affects 100–200 persons per million of population and is a disease defined clinically by the presence of diffuse and extensive skin fibrosis, and is associated with autoimmunity, vascular damage, and progressive fibrosis of internal organs (Jinnin, 2010). It is not known how the autoimmunity, vasculopathy and fibrosis are linked, but vascular dysfunction and autoimmunity are present at the earliest stages of the disease.

Scleroderma vessels have an unusual endothelial phenotype, with a loss of normal markers including vascular endothelial (VE)-cadherin and a gain of fibroblast markers (Fleming et al., 2008). Further comprehensive transcriptional analysis of skin biopsies from SSc patients has demonstrated the presence of inflammation and TGFβ/Smad gene signatures (Cipriani et al., 2015; Manetti et al., 2017). TGFβ can act directly on fibroblasts to induce cell proliferation, migration, and activation and transcription of profibrotic molecules such as collagens and fibronectin. TGFβ also induces the expression of SERPINE1 (PAI-1), which inhibits the action of plasminogen activator, plasmin activator and plasminogen, plasmin that break down ECM components. In addition, TGFβ can drive EndMT to further supplement the population of activated fibroblasts at sites of injury. The massive deposition of ECM proteins leads to fibrosis, the loss of tissue architecture, and ultimately the loss of organ function.

### 4.5 EndMT and atherosclerosis

Atherosclerosis is a complex condition which contributes significantly to pathogenesis of cardiovascular diseases including coronary artery disease, peripheral artery disease, myocardial infarction and stroke. The pathophysiology of atherogenesis involves decades-long expansion of the arterial intima due to the accumulation of lipids, inflammatory molecules, immune cells, SMCs, and ECM, resulting in development of atherosclerotic plaques. As these plaques grow in size they can induce lumen narrowing while their rupture and subsequent thrombosis can result in an abrupt vessel closure with potentially fatal consequences (Tabas et al., 2015).

While the growth of atherosclerotic plaques has been thought to be largely driven by a combination of hyperlipidemia and chronic vascular inflammation, key details of this process remained poorly understood. Recently, EndMT has been proposed as a major factor responsible for the initiation and progression of atherosclerotic plaques growth (Schwartz et al., 2018). This presents an exciting new avenue in understanding atherosclerosis disease genesis and progression. A study from our laboratory was the first to use a specific EC Cre driver to trace the origin of cells in developing plaques in mice carrying a deletion of the ApoE gene. The results showed a very extensive EndMT with over 95% of lineage-traced ECs in atherosclerotic lesions expressing mesenchymal or SMC markers (Chen et al., 2015). The significance of this result was further corroborated in studies of human atherosclerotic plaques. As measured by phospho-Smad2 staining, a readout of the TGFβ activity, more than 90% of ECs from sections of atherosclerotic human coronary artery plaques showed activated TGFβ signaling and increased expression of SMC (Notch3, SM22α), mesenchymal (collagen 1, fibronectin), and adhesion molecules (ICAM-1, VCAM-1) markers expression in luminal endothelial cells. Importantly, there was a strong correlation between the extent of endothelial TGFβ signaling activation, the extent of EndMT and the severity of atherosclerosis (Chen et al., 2015). These finding were supported by the study of Evrard et al. who confirmed the presence of EC-derived mesenchymal cells using endothelial-SCL-Cre-YFP lineage tracing in hyperlipidemic mice (Evrard et al., 2016). The authors were able to show that not only EndMT contributes to atherosclerotic lesions development but is associated with plaque instability. Another important factor contributing to atherosclerosis is the disturbed fluid shear stress in certain areas of the vascular system, particularly curvatures and branch points. These areas demonstrate early onset of inflammation and plaque development with overlaying ECs exhibiting increased levels of nuclear phospho-Smad2 (Hahn and Schwartz, 2009; Moonen et al., 2015).

While these observational studies strongly suggested a causal association of EndMT and atherosclerosis, the direct mechanistic linked was demonstrated in the study of Chen et al. (2019) using an inducible, EC-specific deletion of TGFβR1 and TGFβR2 genes (Chen et al., 2019). When induced prior to the initiation of hyperlipidemia, this endothelial-specific disruption of TGFβ signaling strongly inhibited the development of atherosclerosis. Critically, when induced after the development of atherosclerotic plaques was fully established, this induced propound (∼60% over 2 months) regression of these lesions. These results conclusively demonstrate that EndMT the key important driver responsible for the development and maintenance of atherosclerotic lesions.

In practical terms, any initial therapeutic interventions aimed at EndMT would be targeted to patients with severe, established disease and their goal would be a reduction in plaque size and its stabilization. Importantly, any anti-TGFβ therapy would have to endothelium-targeted as systemic inhibition of TGFβ signaling would result in severe side effects. The feasibility of this approach has been recently demonstrated using endothelial specific lipid nanoparticle-based delivery of TGFβR1/R2 siRNAs (Chen et al., 2019).

### 4.6 EndMT and pulmonary hypertension

Pulmonary Hypertension (PH) is defined as a sustained increase of blood pressure in the pulmonary circulation to more than 25 mmHg at rest or 30 mmHg with physical exertion (Simonneau and Hoeper, 2019). It affects mainly young to middle aged women and survival rate is estimated at 55%–65% at 3 years post diagnosis. There is no cure for this disease except for the treatment of the symptoms. Understanding the mechanisms contributing to PH might give us an opportunity to develop potential therapeutics. Pulmonary artery remodeling is a major feature of PH. It is characterized by cellular and structural changes affecting all three layers of pulmonary artery vessel wall. In addition, increased production of the ECM including extensive deposition of collagen and elastin, contribute to lumen narrowing of pulmonary arterial vasculature leading to reduced blood flow, increased pulmonary arterial pressures, right heart failure and, ultimately, death (Lai et al., 2014).

Two recent papers reported the occurrence of EndMT in PH; Ranchoux et al. showed in both human and animal samples the cellular co-localization of VE-cadherin and SM α-actin, while Good et al. observed that 5% of lung cells in pulmonary hypertension patients were positive for both vWF and SM α-actin (Good et al., 2015; Ranchoux et al., 2015). Furthermore, a knockout of endothelial TGFβR1 in mice with MEKK3-deletion dependent PH resulted in prevention of PH development (Deng et al., 2021). These studies highlighted the importance of the TGFβ signaling and focused future studies to investigate this pathway in more detail.

### 4.7 EndMT and vasculitis

Vasculitis is a group of diseases characterized by inflammation in and around blood vessels affecting various organs such as the kidneys, skin, joints and lungs, among others. Vasculitis can affect both adults and children and the disease spectrum can range from mild and transient to life-threatening. The etiology of most vasculitis syndromes is unknown. In the anti-neutrophil cytoplasmic autoantibody (ANCA)-associated vasculitis, ANCA bind to activated neutrophils and trigger neutrophils release neutrophil extracellular traps (NETs). NETs contain proinflammatory proteins and are thought to contribute to vessel inflammation directly by damaging ECs (Schreiber et al., 2017). A recent study show that NETs can induce EndMT (Pieterse et al., 2017). These findings provide a foundation for future possible anti-EndMT vasculitis therapeutic exploration.

### 4.8 EndMT and Friedreich’s Ataxia

Friedreich’s ataxia (FA) is an inherited disorder characterized by progressive neurodegeneration, limb muscular weakness, and cardiomyopathy. It is caused by a genetic defect in a mitochondrial protein, frataxin. Frataxin reduction results in decreased production of adenosine triphosphate (ATP), abnormal iron accumulation, generation of reactive oxygen species, increased oxidative stress, and ultimate cell damage (La Rosa et al., 2020). While FA is best known for its association with progressive neurodegeneration, heart disease is the most common cause of death in this patient population (Hanson et al., 2019). Fibroblast activation and excess deposition of the extracellular matrix in the cardiac muscle by activated fibroblasts play critical roles in pathogenesis and progression of cardiac fibrosis in FA patients. Importantly, EndMT is present in heart samples obtained from FA patients (Koeppen et al., 2020), suggesting that EndMT may contribute to cardiac fibrosis. It would be interesting to explore the pathophysiological importance of EndMT in the context of vascular inflammation and cardiac fibrosis seen in FA patients.

## 5 Transcription factors regulating SMC phenotypic modulation in coronary artery disease

Coronary artery disease (CAD) is a common disease caused by a buildup of plaques in coronary arteries, blood vessels that bring oxygen-rich blood to the heart muscle. The most important pathological condition causing CADs is atherosclerosis (Ross, 1999), a progressive process that, over decades, leads to accumulation of lipids, fibrous elements and chronic inflammation in the vessel wall leading to formation and growth of atherosclerotic plaques. Among many pathologic features of this process, and hence of CAD, are the loss of the normal barrier function of the endothelium, lipoprotein accumulation in the blood vessel wall, recruitment of monocytes and lymphocytes to the artery wall and SMC proliferation and migration to populate neo-intimal lesions (Hansson, 2005). CAD is caused by both environmental factors and genetic changes (Marenberg et al., 1994). Defining genes that affect CAD risk and understanding their mechanisms may help to improve its prevention and treatment.

SMCs are key contributors to the stability of the atherosclerotic fibrous cap. Medial SMCs undergo partial cell fate transition to become ECM-secreting fibromyocytes, a cell type similar to fibroblasts. Fibromyocytes then proliferate, migrate, and secrete abundant levels of ECM to stabilize the fibrous cap (Wirka et al., 2019). Given the significant contribution of SMC phenotypic modulation to CAD etiology, it is not surprising that genes related to this process contribute to CAD heritability. Genome-wide association studies (GWAS), a widely used technique in human genetics research to find DNA variations associated with common human diseases, identified several novel loci associated with CAD (Nikpay et al., 2015). Among those, TCF21 (Transcription Factor 21) and SMAD3 are two examples of CAD causal genes. These two transcription factors have opposing functional roles in regulating SMC phenotypes (Schunkert et al., 2011; Erdmann et al., 2018; Liu et al., 2018).

SMAD3 gene encodes a transcription factor critical to promotion of SMC differentiation (Figures 2C, D). The human SMAD3 gene is located on chromosome 15 (15q23). It spans over 130 kb and consists of 9 exons in total. Most of SMAD3 exons are clustered towards the 3’ end of the gene, with a large intron almost 100 kb in size separating exons 1 and 2 (Turner et al., 2016). Turner and co-workers have identified a novel CAD-associated SNP rs17293632C>T in the SMAD3 locus (Turner et al., 2016). SMAD3 rs17293632 SNP affects a consensus AP-1 (activator protein 1) transcription factor binding site in the SMAD3 intron 1 enhancer, reducing the enhancer’s activity and, as a result lowering SMAD3 expression (Turner et al., 2016). Importantly, *in vivo* experiments in mice further confirmed these findings: a recent study from Prof. T. Quertermous’ lab showed that SMAD3 is the CAD functional gene and determined the gene regulation network that mediates this causality (Cheng et al., 2022).

Genetic variants in TCF21 are also associated with CAD risk. TCF21 antagonizes Smad3-mediated gene expression and promotes SMC dedifferentiation, proliferation, and migration. Reduced TCF21 expression upregulates SMC differentiation markers (Iyer et al., 2018). At the molecular level, TCF21 interferes with the SRF/Myocardin signaling cascade to promote SMC dedifferentiation. TCF21 binds to CArG boxes of contractile markers to reduce SRF/Myocardin mediated transactivation (Nagao et al., 2020). In regard to atherosclerosis, TCF21 is induced early during atherosclerosis development to suppress SMAD3-mediated gene expression and allow SMCs to switch to a fibromyocyte phenotype. SMC specific Tcf21 knockout results in a thin fibrous cap in mouse models (Wirka et al., 2019). This effect was confirmed by human genetics where the CAD rs12190287 risk allele was associated with reduced TCF21 expression (Miller et al., 2013).

### 5.1 Transcription factors regulating EC and SMC cross-talk in atherosclerosis

An important feature of atherosclerosis is that atherosclerotic plaques do not form uniformly throughout the arterial vasculature. The plaques have a predilection for regions of disturbed shear stress such as the lesser curvature of the aorta, and bifurcation and branching sites (Chiu and Chien, 2011) termed atheroprone regions. In contrast, segments of arterial vasculature with high, steady laminar shear stress tend to remain atherosclerosis-free due to activation of the atheroprotective endothelial transcriptional program. In atheroprotective regions, laminar shear stress activates endothelial surface mechanosensors leading to MEF2 (myocyte enhancing factor-2) translocation to the nucleus and binding to KLF4 promoter region which causes suppression of inflammation (Wang et al., 2013; Maejima et al., 2014; Baeyens et al., 2015; Lu et al., 2021; Tanaka et al., 2021). At the same time, shear stress acting on EC MEF2/KLF4 axis can influence SMC mobility by regulating neuronal guidance molecule, Sema3d (Lu et al., 2017). *In vivo* experiments in mice with Mef2c deletion in ECs show increased atherosclerosis which is caused by a profound increase in medial SMC migration. Small number of the medical SMCs may undergo selective clonal expansion and may end up being the dominant disease-prone subpopulation of SMCs within the neointima (Chappell et al., 2016). MEF2 and KLF4 confer anti-inflammatory and anti-migratory properties to ECs and SMCs, therefore positioning these transcription factors as an interesting target for therapeutic interventions.

### 5.2 TGFβ-mediated transcription in aortic aneurysm development

Aortic aneurysm can occur in a number of conditions ranging from MFS and related syndromes to aging, injury, inflammation and atherosclerosis. In all cases, there is a weakening and disruption of the aortic wall that directly leads to aneurysm formation. SMC have been considered an important cell type involved in aneurysm development but how these cells contribute to this process had not been well understood.

An aneurysm is defined as a 50% localized increase in the aortic diameter (El-Hamamsy and Yacoub, 2009). They can occur anywhere along the length of the aorta and are categorized into two main groups depending on their location: thoracic aortic aneurysms (TAA) and abdominal aortic aneurysms (AAA). While TAA have a strong genetic basis, AAA are associated with advanced age, male sex, and atherosclerosis in combination with the usual cardiovascular risk factors (e.g., hypercholesterolemia, hypertension, and/or diabetes). During the past two decades, it has become clear that most sporadic TAA identified in patients over age 65 years share many of the lifestyle-associated risk factors as AAA including age, sex, smoking, hyperlipidemia, and hypertension although there are important differences between the two syndromes. To date, there are no effective treatments that can slow or reverse aneurysm growth.

**FIGURE 5:**
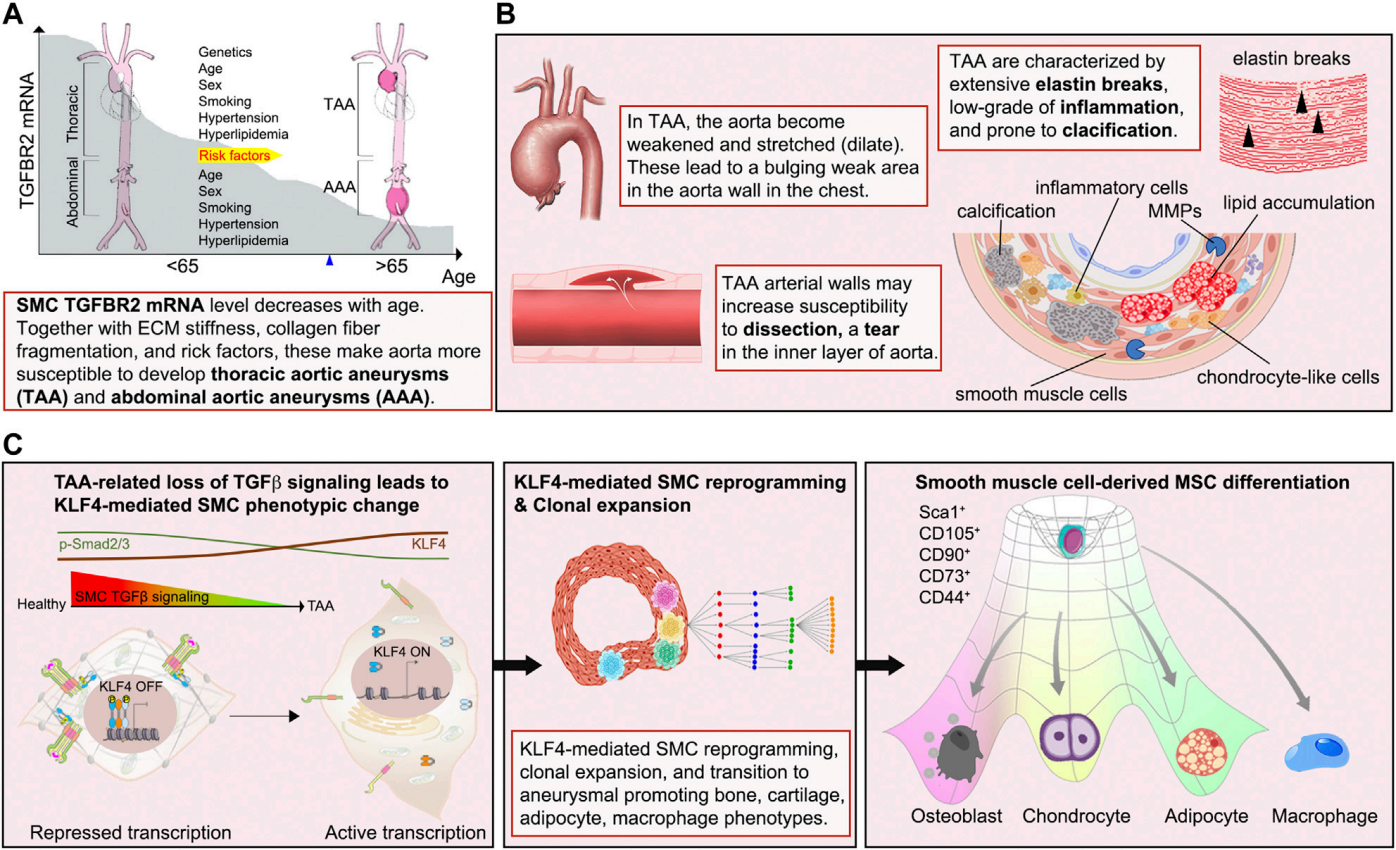
Diagram illustrating the sequence of events of aortic aneurysm development. **(A, B)** Externally or internally triggered inflammation and/or injury of the vessel wall can induce fate transitions of normal SMCs leading to potentiation of inflammation via production of pro-inflammatory molecules, the appearance of SMC-derived bone- and cartilage-producing cells as well as macrophage-like cells, leading to disorganization of the aortic extracellular matrix. These changes result in the weakening of the aortic wall, resulting in its dilation and aneurysm formation. **(C)** Events responsible for calcification, elastin breaks, and inflammation of the TAA include i) upregulation of the stem cell pluripotency gene KLF4 due to the loss of TGFβ signaling input, ii) reprogramming of SMC to mesenchymal stem cell (MSCs) state by KLF4, iii) clonal expansion of a disease-prone subsets, iv) tri-lineage differentiation of MSCs into osteoblasts-, chondrocytes-, adipocytes-like cells, and appearance of SMC-derived macrophage-like cells.

An important feature of aortic aneurysm is their association with chronic aortic wall inflammation, increased local expression of proteinases, excessive collagen deposition, and elastolysis. Immunocytochemical analysis of human TAA tissues shows decreased TGFBR2 mRNA expression and increased elastic fiber fragmentation in SMCs of the aortic media. There is also an accumulation of lipid, leukocytic infiltration and calcifications (Chen et al., 2020) (Figures 5A, B). These processes lead to weakening of the aortic wall, leaving it prone to dissection. Recent evidence suggests that SMC phenotype switch is one of the primary underlying defects leading to aneurysm formation. A conditional SMC-specific knockout of Tgfbr2 in mice with hyperlipidemia (TGFβR2^iSMC−Apoe^) closely mimics development of human aortic aneurysms (Chen et al., 2020). Immunocytochemical lineage tracing analysis of aortic tissues harvested from these animals revealed that the presence of large numbers of cells in the aortic media expressing chondrocyte (aggrecan), osteoblast (osteopontin), adipocyte (adiponectin) and macrophage (Mac2) markers that were of SMC origin thus suggesting the presence of extensive SMC-to-mesenchymal transition (Chen et al., 2020).

A causal link between suppression of SMC TGFβ signaling and aneurysms development was further confirmed using sequential single cell sequencing analysis combined with Imaging Mass Cytometry. Analysis of the time course of changes in gene expression in various SMC subsets revealed that a single SMC population present in the aortic SMCs at the onset of TGFβ signaling disruption accounted for essentially all SMC-derived bone, cartilage, adipocyte and macrophage cell lineage (Figure 5C). The idea of a single SMC population expansion accounting for most of the observed pathology was further confirmed by studies using multi-color R26R-Confetti reporter mice that clearly showed the clonal nature of SMC-derived bone, cartilage, adipocyte, and macrophage populations in the aortic aneurysm (Chen et al., 2020) (Figure 5C). These observations point to the existence of disease-prone and disease-resistant SMC populations in the normal aortic wall and suggest that increased presence of disease-prone SMCs may account for higher frequency of aortic aneurysms in specific patient populations.

SMC reprogramming plays a major in the observed cell fate changes. ChIPseq transcription studies identified KLF4 as a key transcription factor mediating cellular SMC reprogramming towards multipotency. Critically, disruption of KLF4 expression in SMC of TGFβR2^iSMC−Apoe^ mice resulted in a significant decrease in aneurysm development (Chen et al., 2020). These comprehensive studies clearly demonstrate that a disease-prone subpopulation SMCs can reprogram into multiple like-cell types that can then induce the loss of aortic structural integrity and aneurysm formation.

## 6 Therapeutic targeting of the TGFβ signaling pathway in human diseases

**FIGURE 6:**
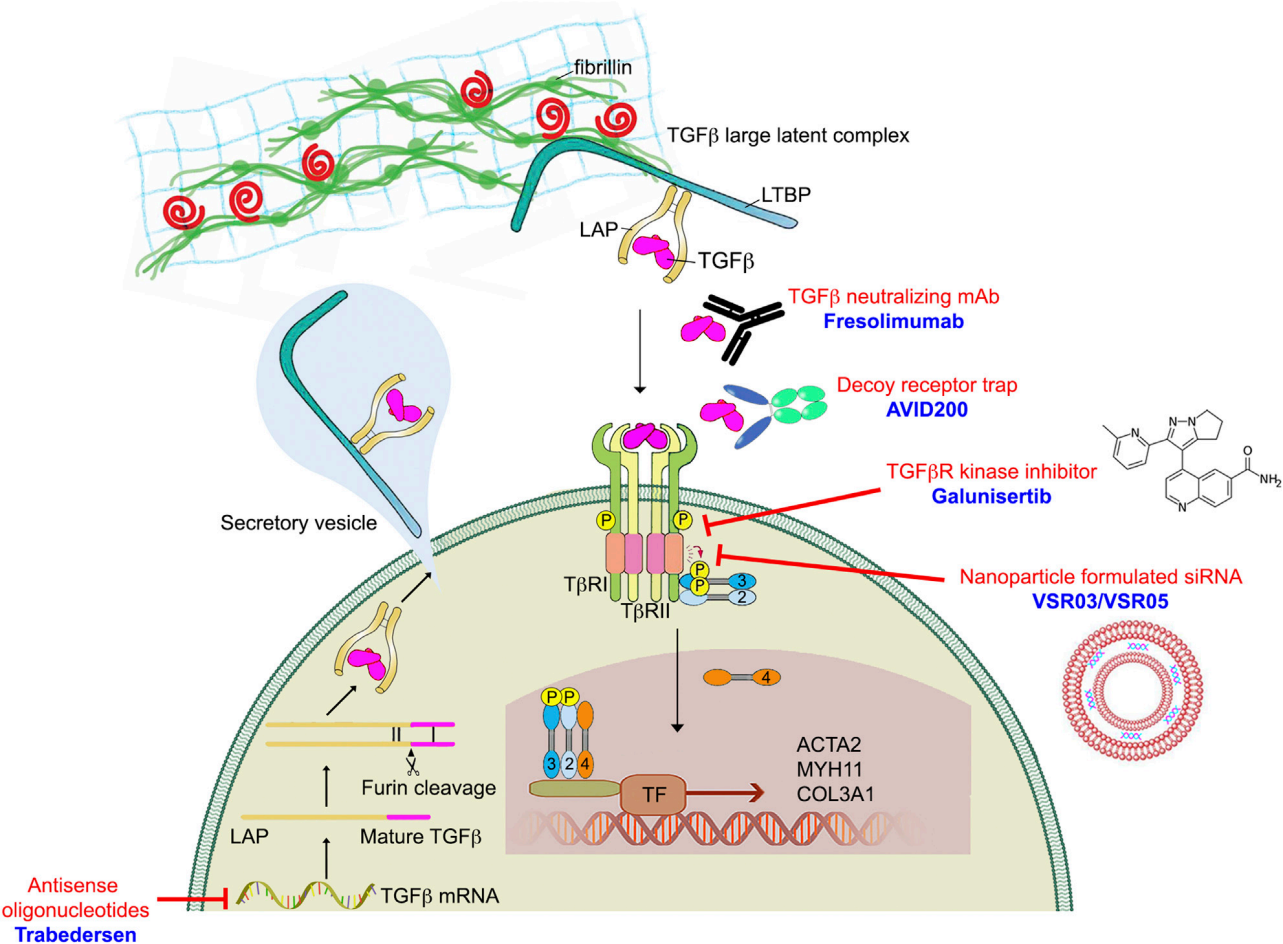
TGFβ blocking drugs in preclinical and clinical trials.

Given the importance of TGFβ signaling in a range of human diseases, academic laboratories and pharmaceutical companies around the world have developed various TGFβ signaling blocking strategies at multiple levels along the TGFβ signaling pathway (Figure 6). These approaches include anti-ligand antisense oligonucleotides (ASOs), antibodies, ligand-competitive peptides, and small-molecule inhibitor (SMIs). Although TGFβ inhibition has proven remarkable efficacy in various preclinical models, translation of these results into the clinic has been disappointing. In this section, we will first discuss these various TGFβ blocking approaches then discuss what are some of the clinical challenges facing anti-TGFβ therapies.

### 6.1 TGFβ ligand targeting

Antisense oligonucleotides (ASO) were the first RNA-based therapeutic that were developed to regulate gene expression (Simons et al., 1992). They were designed to be 13–25 nucleotides long, single stranded, and to bind complementary sequences of target mRNA via Watson-Crick hybridization. This induces target endogenous mRNA degradation and translational repression (Askari and McDonnell, 1996). Unmodified ASOs have a low efficacy and are degraded quickly by nucleases in the blood and in tissues. Although chemical modifications of ASOs can increase their stability but the efficiency of this approach remains fairly low. One example is Trabedersen (AP12009, Isarna Therapeutics), an 18-mer synthetic antisense phosphorothioate oligodeoxynucleotide targeting human TGFB2 mRNA for treatment of malignant glioma, pancreatic carcinoma, and malignant melanoma (Jaschinski et al., 2011). Although Phase I and Phase II trials of Trabedersen looked promising, the multinational Phase III study was terminated due to significant side effects and insufficient targeted delivery of antisense oligonucleotide. This result prompted greater focus on developing various delivery methods to improve ASO efficiency. Trabedersen is currently not under further clinical development.

### 6.2 TGFβ ligand-receptor interaction targeting

Several studies have employed ligand traps (monoclonal antibodies and soluble receptors) and anti-receptor monoclonal antibodies to block TGFβ-dependent signaling activation. Eli-Lilly & Co developed a humanized monoclonal antibody LY2382770 which selectively targets TGFβ1. However, Phase II dosing studies in diabetic kidney disease were terminated due to the lack of efficacy (Voelker et al., 2017). Another TGFβ1-neutralizing antibody, CAT-192 (Cambridge Antibody Technology (CAT) and Genzyme Corp), did not show evidence of efficacy in Phase I/II clinical trials for early-stage systemic scleroderma (Denton et al., 2007). In addition to selective antibodies that neutralize TGFβ1, pan-anti-TGFβ antibodies have also been developed and evaluated in fibrotic diseases. In particular, Fresolimumab (GC1008), a humanized monoclonal antibody in the Sanofi pipeline, neutralizes all three TGFβ isoforms (TGFβ1, TGFβ2, and TGFβ3). Fresolimumab was safe and well tolerated in both Phase I and II clinical trials but failed to demonstrate efficacy in treatment-resistant primary focal segmental glomerulosclerosis (Trachtman et al., 2011; Vincenti et al., 2017).

The excess of TGFβ production in the tumor and fibrosis microenvironment can also be controlled by decoy receptors. These molecules inhibit TGFβ signaling by competing with TGFβ receptors for ligand binding. One such TGFβ trap is AVID200, a computationally-designed TGFβ1/3 ligand trap developed by Forbius (Varricchio et al., 2021). AVID200 was constructed by fusing TGFβR ectodomains to a human IgG Fc region. TGFβ2 plays a key role in normal cardiac function and thus an undesirable drug target (Sanford et al., 1997; Bartram et al., 2001). Importantly, Forbius claims that AVID200 does not hit TGFβ2, which is crucial to minimize toxicities. AVID200 is currently in Phase I trials for fibrosis and solid tumors.

### 6.3 TGFβ receptor kinase inhibitors and peptide aptamers

Small-molecule inhibitors (SMIs) are an important and widely used class of medications. They can be produced cost-effectively on a large scale and have the advantage of being taken orally, unlike peptide drugs, antibodies and ASOs. Based on the crystal structure of the TGFBR1-FKBP12 complex, both Eli-Lilly & Co and GlaxoSmithKline began their own medicinal chemistry program searching for TGFβR1 inhibitors via high throughput screens. Galunisertib (also known as LY2157299), developed by Eli-Lilly & Co, has shown some clinical benefit in various types of cancers, however research has identified serious cardiotoxic effects associated with continuous administration of TGFβR1 SMIs (Herbertz et al., 2015). GlaxoSmithKline stopped development of its TGFβR1 SMI program due to heart valve damage concerns. AstraZeneca also developed two TGFβR1 kinase inhibitors, AZ12601011 and AZ12799734. Their preclinical studies showed both inhibitors have off-target inhibition of the TGFβ family bone morphogenetic protein (BMP) receptors and cardiovascular toxicity in rats (Anderton et al., 2011; Spender et al., 2019).

An alternative approach that promises more specific disruption of TGFβ/Smad-dependent signaling is the use of peptide aptamers. Trx-SARA is an example of such an aptamer, which is made of a rigid scaffold Trx (the *Escherichia coli* thioredoxin A protein) linked to the Smad-binding domain of SARA (a constrained 56-amino acid Smad-binding motif from the SARA protein) (Zhao and Hoffmann, 2006). Trx-SARA binds specifically to Smad2 and Smad3 and inhibits TGFβ induced gene expression. No clinical trials have been undertaken with peptide aptamers.

### 6.4 Nanotechnology-based therapeutic drug delivery

Not surprisingly, none of the candidate drugs mentioned above have made to approval for any clinical indication. In all cases, either a lack of efficacy or safety concerns were the overriding considerations. The main reason is that TGFβ effects, while biologically and clinically important, are highly cell type- and context-dependent. Therefore, a systemic TGFβ inhibition while achieving a desirable effect on one set of cells or tissues is almost certain to have deleterious effects on other cells and tissues. One illustration of these difficulties is a recent RNAseq study of TGFβ signaling effects in endothelial vs. smooth muscle cells vs. macrophages (Chen et al., 2019). While TGFβ treatment was anti-inflammatory in SMCs, it was pro-inflammatory in ECs (Chen et al., 2019). Furthermore, in the case of the vasculature, mouse studies have demonstrated that while TGFβ signaling disruption is anti-atherosclerotic in ECs, it is pro-atherosclerotic in SMCs, T cells, macrophages, and dendritic cells (Robertson et al., 2003; Reifenberg et al., 2012; Lievens et al., 2013; Chen et al., 2019; Chen et al., 2020) (Table 2).

**TABLE 2:** Summary of tissue specific TGFβ signaling inactivation in mouse genetic hyperlipidemia mouse models.

Cell type	Gene	Disease model	Phenotype	Reference
Endothelial cell (Cdh5Cre)	Tgfbr1/Tgfbr2 knockout	Hyperlipidemia	Reduced atherosclerosis progression and pronounced plaque regression	Chen et al. (2019)
Smooth muscle cell (Myh11Cre)	Tgfbr2 knockout	Hyperlipidemia	Enhanced atherosclerosis and induction of aneurysm formation	Chen et al. (2020)
Macrophage SRA (scavenger receptor A)	Tgfb1 overexpression	Hyperlipidemia	Reduced atherosclerosis	Reifenberg et al. (2012)
Dendritic cell (CD11c)	Dominant negative Tgfbr2 overexpression	Hyperlipidemia	Enhanced atherosclerosis	Lievens et al. (2013)
T cell (CD4)	Dominant negative Tgfbr2 overexpression	Hyperlipidemia	Enhanced atherosclerosis	Robertson et al. (2003)

These considerations suggest that the only practical approach to therapeutic manipulation of TGFβ signaling would have to involve cell type specific targeting. Recent developments in nanotechnology-based drug delivery are becoming a new paradigm for cell-targeted therapies. Among various classes of nanoparticles, lipid-based nanoparticles (LNPs) are the most clinically tested non-viral drug delivery systems. In particular, LNPs have been used since the mid-2000s for deliver RNA therapeutics. Researchers from Alnylam Pharmaceuticals Inc., and Protiva Biotherapeutics Inc., were able to use a LNP to systemically deliver a small double-stranded RNA fragments (siRNA) to non-human primates (NHPs) (Zimmermann et al., 2006; Akinc et al., 2008). Over the last few years, LNP-based RNA vaccines have been administered to hundreds of millions of people.

These nanoparticles are made of a polyethylene (PEG) lipid, phospholipid, cholesterol, and a cationic lipid and have an average particle size of 70–90 nm and are suitable for delivery of DNA and RNA molecules (Brader et al., 2021; Cui et al., 2022). When loaded with RNA, LNPs not only protect their payload from degradation but are also capable of delivering it to cells and facilitate their intracellular entry (Whitehead et al., 2009). When injected intramuscularly or intravenously, LNPs deliver most of their payload to the liver where a dose as low as 2.5 mg/kg can effectively suppress hepatocyte gene expression for as long as 11 days after a single injection (Zimmermann et al., 2006).

While to date all clinically approved LNP-base therapies target the liver (Love et al., 2010), there is an urgent need to develop LNP formulations that allow payload delivery to non-hepatic organs and tissues. This has been, in part, facilitated by the development of new lipid-like molecule lipidoid libraries (Love et al., 2010; Alabi et al., 2013). By screening epoxide-modified lipid-polymer hybrids at different lipid:siRNA ratios, R. Langer, D. Anderson and colleagues identified a new branched lipid compound termed 7C1 that exhibit long circulation time and preferential payload delivery to ECs with reduced hepatic accumulation (Dahlman et al., 2014). Interestingly, the reason for the preferential endothelial uptake of 7C1 LNPs has never been established. Nevertheless, a number of studies using 7C1 LNPs have demonstrated effective and endothelial-specific targeting (Jung et al., 2017; Sago et al., 2018a; Sago et al., 2018b; Khan et al., 2018; McCann et al., 2019; Park et al., 2019; Yu et al., 2019; Culley et al., 2021; Lee et al., 2022) and this approach has proved to be effective in treatment of atherosclerosis in mice as discussed below (Chen et al., 2019).

RNA-based drugs offer unique opportunities to expand the range of therapeutic targets in addition to small-molecule inhibitors and protein-based drugs. RNA interference (RNAi) is a conserved biological process that cells use to suppress the activity of specific genes. It is mediated by small double-stranded RNA fragments (siRNA) that play a key role in gene regulation in the intrinsic defense against RNA virus and transposon (Fire et al., 1998). The discovery of RNA-mediated gene silencing has given researchers unprecedented opportunities to develop novel therapeutic agents. siRNA drugs have many advantages over existing small molecule or monoclonal antibody-based therapies, including (1) simple manufacturing; (2) ease of modifications to improve potency and stability; (3) potential to target any transcript in the cell.

MicroRNAs (miRNAs) provide another interesting therapeutic option. miRNAs were originally discovered in 1993 by V. Ambros during a study of the gene lin-14 in the development of *C. elegans* (Ambros, 2004). There are numerous miRNAs that show a striking degree of conservation in plants and animals, but not in bacteria (Tjaden et al., 2006). miRNAs are a class of short non-coding RNAs (∼21–23 nucleotides) (Ambros, 2004). They do not encode proteins but are capable of negative regulation of gene expression at a post-transcriptional level. Computational predictions indicate that more than 50% of all human protein - coding genes are potentially regulated by miRNAs (Lewis et al., 2005). Many miRNAs targeting TGFβ signaling components have been identified. For examples, the let-7 family targets TGFβR1 and Smad2 and the miR-200 family are able to inhibit the expression of TGFβ, TGFβR1, and Smad2 (Braun et al., 2010; Chen et al., 2012; Liao et al., 2014; Gregory et al., 2011). miR-29 has been shown to target Smad3 and collagen (Li et al., 2009; Leone et al., 2012; Gallant-Behm et al., 2019). miR-21 and miR-182 target Smad7 and thus, modulates a negative feedback loop of TGF-β signaling (Li et al., 2013; Yu et al., 2016). The aberrant expression of the miRNAs has also been implicated in a number of cardiovascular pathologic settings. For example, let-7 miRNA family is highly expressed in the cardiovascular system and is downregulated in certain disease setting such as cardiac hypertrophy, cardiac fibrosis, dilated cardiomyopathy, and myocardial infarction (Bao et al., 2013).

Like siRNA, using miRNAs as therapeutic targets may well become an important tool in the development of new therapeutics. Mature miRNAs have many advantages as a therapeutic modality, including their conserved sequences across species and the ability to target multiple components withing a signaling network. Agomirs and antagomirs can be used to regulate miRNAs expression. Agomirs are double-stranded nucleotide sequences that mimic endogenous miRNAs and regulate biological functions of the target gene. On the other hand, antagomirs are single-stranded and designed in a way that they can bind to the mature miRNA, inhibiting its expression (Krutzfeldt et al., 2005). Since RNAs are extremely susceptible to serum nucleases, a typical agomir or antagomir has chemical modifications to enhance nuclease stability and target binding affinity. The major types of chemical modifications that are being investigated in the preclinical setting are phosphorothioate (PS), ribose 2’-OMe backbone modifications and locked nucleic acid modification (Cummins et al., 1995; Pfundheller and Lomholt, 2002; Eckstein, 2014).

While the RNA-based therapy is promising, *in vivo* RNA drug delivery is very challenging. The RNA drugs must avoid triggering immune response, escape from phagocytosis in the bloodstream, reach the target tissue, and get into target cells. To address these RNA drug delivery challenges, Alnylam Pharmaceuticals Inc. together with partners at MIT developed novel delivery methods of chemically modifying siRNAs encapsulated in LNPs. In 2018, the US FDA approved the first LNP-based siRNA drug Onpattro (patisiran) for the treatment of hereditary transthyretin amyloidosis (hATTR) (Kristen et al., 2019). Since then, three additional siRNA-based therapies (givosiran, lumasiran, and inclisira) have been approved by US FDA (Garrelfs et al., 2021; Kallend et al., 2022; Ventura et al., 2022).

As discussed earlier, targeting TGFβ signaling may have therapeutic benefits in CVD treatment. Such targeting may lead to reprogramming of EndMT, reducing its inflammatory influence over vessel wall. Based on these considerations, we employed 7C1 LNP-based delivery of siRNA targeting TGFβ receptors in ECs for the treatment of atherosclerosis (Chen et al., 2019). This means of delivery resulted in preferential uptake of anti-TGFβ receptors siRNAs by the endothelium with little to no delivery to SMCs and macrophages (Chen et al., 2019). When given at the time of atherosclerosis induction to ApoE null mice, 7C1-based delivery of Tgfbr1/2 siRNAs resulted in suppression of atherosclerotic plaque growth with virtually no inflammation, characteristic of this model, evident in the arteries (Chen et al., 2019). Most promisingly, when delivered to animals with established atherosclerotic lesions, 7C1 delivery of Tgfbr1/2 siRNA led to ∼60% reduction in the size of the plaques (Chen et al., 2019). The endothelium is critically involved in various diseases, researchers around the globe have used 7C1 LNP delivery platform for various cancer, CVD, and neuromuscular disease model treatments (Dahlman et al., 2014; McCann et al., 2019; Yu et al., 2019; Culley et al., 2021).

## 7 Conclusion and perspectives: the TGFβ paradox

Marfan syndrome has historically been seen as a prototypical disease associated with thoracic aortic aneurysms (TAA). The development of TAA in Marfan’s setting has been linked by H. Dietz and others to activation of TGFβ signaling due to, in part, higher levels of circulating TGFβ that were shown to positively correlate with aortic root diameter and have therefore been proposed as biomarker for aortic risk. In line with this, high levels of Smad2/3 activity and its downstream TGFβ-responsive targets were found in human aneurysm tissues and mouse aneurysm models. Critically, these *in vivo* studies were performed using conventional global knock-ins mimic MFS (*Fbn1* ^C1039G/+^) and LDS (*Tgfbr1* ^*M318R/+*^ and *Tgfbr2* ^*G357W/+*^) mouse aneurysm models that ignored essential cell non-autonomous cell-cell interactions and communication effects of the TGFβ pathway (Holm et al., 2011; Gallo et al., 2014). As the result of these studies, clinical trials using the Angiotensin II receptor blocker losartan were carried out in MFS patients. Losartan was believed to inhibit TGFβ signaling and reduce the progression of aortic aneurysms (Habashi et al., 2006). However, the evidence for this treatment is relatively weak and conflicting results have been reported between the initial studies and further larger randomized clinical trials (Brooke et al., 2008; Lacro et al., 2014).

Recent mouse genetic evidence has yielded strikingly opposite results regarding the role of TGFβ signaling in aortic aneurysms. Studies in mice with SMC-specific Tgfbr2 knockout showed a downregulation of SMC marker gene expression, loss of SMC contractile properties correlated and the development of thickened, dilated aortic wall as well as frequent thoracic aorta disections (Li et al., 2014; Hu et al., 2015). These observations are supported by studies implicating downregulation of TGFβ signaling in the regulation of SMC cell fate (Chen et al., 2020). Histological Verhoeff-van Gieson staining of aortas in mice with conditional SMC-specific knockout of Tgfbr2 and hyperlipidemia (TGFβR2^iSMC−Apoe^) shows extensive destruction of the aortic wall and the development of aneurysms bearing many hallmarks of human TAAs. These include degradation of elastic lamellae arrangement, increased atherosclerosis lesion burden, and calcification of the aortic wall (Chen et al., 2020).

Overall, there is currently conflicting evidence for pathogenic versus vasculoprotective role of TGFβ in aortic aneurysms. Genetic evidence in mice is strongly suggestive of a vasculoprotective effect of TGFβ signaling in smooth muscle cells while it is pathogenic in the endothelium (Chen et al., 2019; Chen et al., 2020). A better understanding of the molecular basis of aneurysm development is the first and essential step to the development of preventive and therapeutic strategies for this deadly disease.

In conclusion, many human diseases arise from either inappropriate activation or inhibition of TGFβ signaling pathways. Since the discovery of the TGFβ cytokine 30 years ago, many research groups all over the world have contributed to understanding the molecular biology of this signaling pathway. However, our current knowledge of how TGFβ signaling components work together to regulate specific gene expression and achieve various biological functions in different cell types and tissues remains limited. There are still substantial knowledge gaps in our understanding of the role of TGFβ signaling in disease pathogenesis that need further exploration. In large measure these related to cell type-and context-specific regulation of TGFβ signaling and its consequences. A detailed understanding of these events may allow development of tailored therapeutic approaches to some of the most intractable diseases.

## Abbreviations

MFS. Marfan syndrome; LAP, latency-associated peptide; LTBP, latent-TGFβ-binding protein; LDS, Loeys-Dietz syndrome; SGS, Shprintzen-Goldberg syndrome; FTAAD, Familial thoracic aortic aneurysm and dissection; vEDS, vascular Ehlers-Danlos syndrome.
